# 

**Published:** 1844-04-01

**Authors:** 


### BIBLIOGRAPHICAL RECORD.

1. Address delivered before the American Society of Dental Surgeons, &c. By Chapman A. Harris, M.D. Baltimore, 1843.

---

2. The American Journal and Library of Dental Science. Published under the auspices of the American Society of Dental Surgeons. &c. No. 1, Vol. IV. New York, 1843. Mr. C. Ash, London Agent.

☞ *In exchange.*

---

3. Natural History, Pathology, & Treatment of the Epidemic Fever at present prevailing in Edinburgh and other Towns, illustrated by Cases and Dissections. By J. R. Cormack, M.D. Churchill, London, 1843. Octavo, pp. 182.

---

4. A Supplementary Report on the Practice of Interment in Towns, &c. By Ed. Chadwick, Esq. Presented to both Houses of Parliament, 1843.

---

5. The Cyclopædia of Anatomy and Physiology. Edited by Robert B. Todd, M.D. F.R.S. Part XXV. London, Sherwood and Co. 1844.

---

6. The Phrenological Journal, &c. No. XXV. New series, Jan. 1844.

---

7. The Retrospect of Practical Medicine and Surgery. By W. Braithwaite, Esq. of Leeds. Vol. VIII. July—Dec. 1843.

---

8. An Introduction to Practical Organic Chemistry, with References to the Works of Davy, Brande, Liebig, &c. Small 8vo, pp. 90. Churchill, London, 1844.

---

9. The Creation: illustrated by Six Engravings on Steel; exhibiting the progressive Advance of Creation through the Six Days, in a Series of Letters from a Father to a Son. By W. G. Rhind. London—S. Bagster and Son, 1844. Pp. 400.

☞ *A pious and useful present.*

---

10. The Anatomy, Physiology, and Pathology of the Teeth, &c. &c. By Paul B.

GODDARD, M.D. and JOSEPH E. PARKER, Dentist. Large quarto, with Plates. Philadelphia, 1844.

---

11. Essay on the Physiognomy of Serpents. By H. SCHLEGEL, Doctor of Philosophy, &c. Translated from the German by THOMAS STEWART TRAIL, M.D. &c. &c. 8vo, pp. 254, with Plates. Maclaghlan & Co. Edinburgh, 1844.

---

12. A Letter to the Governors of the Brighton Infirmary, &c. By a Medical Practitioner. Brighton, King, bookseller. 1843.

---

13. The Medical Examiner. Philadelphia. September to the end of December, 1843. ☞ *In Exchange.*

---

14. Glossology; or the Additional Means of Diagnosis of Disease by the indications and appearances of the Tongue. By BENJ. RIDGE, M.D. &c. Octavo, pp. 84, with Plates. Churchill, London, 1844.

---

15. Diseases of the Lungs from Mechanical Causes, &c. By CALVERT HOLLAND, M.D. Physician to the Sheffield General Infirmary, &c. Octavo, pp. 100. Churchill, 1844.

---

16. On the Nature and Treatment of Tic Douloureux, Sciatica, &c. By HENRY HUNT, M.D. Octavo, pp. 191. Churchill, 1844.

---

17. Report of the Commissioners appointed to take the Census of Ireland for the year 1841. Presented to both Houses of Parliament. Folio, upwards of 700 pages, with numerous Tables, &c. Dublin, 1843.

☞ *This valuable compilation, we believe by Mr. Wilde, will be taken due notice of.*

---

18. Observations on the Proximate Cause of Insanity. By JAMES SHEPPARD, M.R.C.S. Duodecimo, pp. 104. Longman and Co. 1844.

---

19. Transactions of the Medical and Physical Society of Bombay. No. 5, for 1842.

---

20. The New York Journal of Medicine and the Collateral Sciences. Edited by SAMUEL FORRY, M.D. Jan. 1844.

---

21. Anatomical Manipulation; or the Methods of pursuing Practical Investigations, &c. By ALFRED TULK, M.R.C.S. & ARTHUR HENFRY, A.L.S. with Illustrations. Small octavo, pp. 413. J. Van Voorst, 1844.

---

22. Lectures on the Theory and Practice of Midwifery, delivered in the Theatre of St. George's Hospital. By ROBERT LEE, M.D. Fellow of the Royal College of Physicians, &c. Octavo, pp. 558. Churchill, 1844.

---

23. The Medical Student's Guide and Almanac for 1844, containing the latest Regulations of all the Licensing Bodies, &c. 8vo, pp. 180. Renshaw, London—Fannin and Co. Dublin. Price 2s. 6d.

---

24. Elements of Physiology, for the Use of Students, &c. By RUDOLPH WAGNER, M.D. Translated by ROBT. WILLIS, M.D. Part 3, Sensation and Motion. Sherwood and Co. 1844. Price 10s.

---

25. Thoughts on Physical Education, &c. By CHAS. CALDWELL, M.D. Second Edition. Edin. 1844.

---

26. The Medical Examiner. Edited by ROBERT M. HUSTON, M.D. Philadelphia. Vol. VIII. Nos. 1 and 2. 1844.

---

27. Scrofula:—its Nature, Causes, and Treatment, &c. By W. TYLER SMITH, M.B. 8vo, pp. 172. Churchill—London, March, 1844.

---

28. The Principal Offices of the Brain and other Centres. By JOSEPH SWAN. Pp. 31. Longmans, 1844.

---

29. Sketches from the Case-book, to illustrate the Influence of the Mind on the Body, with the Treatment of some important Brain and Nervous Disturbances. By R. FLETCHER, Esq. Surgeon to the Gloucester General Hospital, &c. 8vo, pp. 389.

---

30. Medico-Chirurgical Notes and Illustrations. By R. FLETCHER, Esq.

---

31. Public Health: An Oration delivered on the Seventeenth Anniversary of the London Medical Society. By LEONARD STEWARD, M.D. Octavo, pp. 19. Longman and Co. 1844.

---

32. Fifth Annual Report of the Registrar General of Births, Deaths, and Marriages in England. Quarto, 1843.

33. A complete condensed Practical Treatise on Ophthalmic Medicine. By. EDWARD OCTAVIUS HOCKEN, M.D. &c. Part I. pp. 108. Price 3s. Highley, 1844.

---

34. Practical Manual on the Diseases of the Heart and Great Vessels—a work intended to facilitate and extend the study of these diseases. By F. A. ARAN, Interne of the first class of the Hôtel Dieu, &c. &c. Translated from the French, by W. A. HARRIS, M.D. Philadelphia, 1843.

☞ *This is an excellent epitome of a large and important class of diseases, especially as to diagnosis.*

---

35. Minor Surgery; or Hints on the every day Duties of the Surgeon. By HY. H. SMITH, M.D. Lecturer on Minor Surgery &c. Octavo. Philadelphia, 1843. Barrington and Co.

---

36. On Regimen and Longevity, comprising Materia Alimentaria, National Dietetic Usages, &c. By JOHN BELL, M.D. Lecturer on Materia Medica, &c. 8vo. pp. 420. Haswell and Johnson, Philadelphia, 1842.

---

37. Practical Medicine; illustrated by Cases of the most important Diseases. By JOHN M. GALT, M.D. Octavo, pp. 328. Philadelphia, 1843.

---

38. Supplementary Report of the Sanitary Condition of the Labouring Population of Great Britain, as to the practice of Interments in Towns. By EDWIN CHADWICK, Esq. Barrister at Law. 8vo. 1843.

---

39. Two Essays on the Diseases of the Spine. B[illegible]. STAFFORD, Fellow of the Royal College of Surgeons. 8vo. pp. 92. Longman and Co. 1844.

---

40. On the Conical Cornea. By JAMES H. PICKFORD, M.D. M.R.I.A. 8vo. pp. 35. With Plates. Dublin 1844.

---

41. Der Neue Wasserfreund, &c. Three Numbers, 1843. Von Dr. SCHMITZ, Marienberg, near Boppard, on the Rhine.

---

42. Illustrations of the Theory and Practice of Ventilation, with Remarks on Warming, Exclusive Lighting, and the Communication of Sound. By DAVID BOSWELL REID, M.D. F.R.S. &c. Octavo, pp. 451. Longman and Co. March 1844.

---

43. Cases of Dropsical Ovaria removed by the Large Abdominal Section. By D. HENRY WALNE, Surgeon. 8vo. pp. 66. Longman and Co. 1844.

---

44. The Western Journal of Medicine and Surgery. Edited by Drs. DRAKE, VANDELE and COLESCOTT. Louisville, Kentucky.

---

45. General Report of the Royal Hospitals of Bridewell and Bethlem, and of the House of Occupation, for the Year ending Dec. 1843. Octavo, London, 1844.

---

46. Geology: Introductory, Descriptive, and Practical. By DAVID THOMAS ANSTED, M.A. F.R.S. &c. Professor of Geology, King's College, London. Part I. price 5s. Van Voorst, Feb. 1844.

☞ *This promises to be a most valuable and entertaining work.*

---

47. A History of British Fossil Mammalia and Birds. By RICHARD OWEN, F.R.S. Conservator of the Hunterian Museum. Part I. price 2s. 6d. Van Voorst, Feb. 1844.

☞ *The name of the Author is a sufficient guarantee for the value of the work.*

---

48. Medicines, their Uses and Mode of Administration; including a complete Conspectus of three British Pharmacopœias, &c. By J. MOORE NELIGAN, M.D. Physician to Jervis Street, Hospital, &c. 8vo, pp. 432. Fannin and Co. Dublin, 1844.

---

49. The Dublin Journal of Medical Science. No. 73. March 1844.

---

50. An Essay on the Tongue, its Functional Derangements of the Stomach and Bowels, &c. By ED. WILLIAMS, M.B. Cantab. Pp. 119. Simpkin and Marshall. March 1844.

---

51. Practical Observations on the Prevention, Causes, and Treatment of Curvatures of the Spine, &c. By SAMUEL HARE, Surgeon. Second Edition. Churchill, April 1844.

